# Effects of Classroom Active Desks on Children and Adolescents’ Physical Activity, Sedentary Behavior, Academic Achievements and Overall Health: A Systematic Review

**DOI:** 10.3390/ijerph18062828

**Published:** 2021-03-10

**Authors:** Terry Guirado, Camille Chambonnière, Jean-Philippe Chaput, Lore Metz, David Thivel, Martine Duclos

**Affiliations:** 1 Laboratory of the Metabolic Adaptations to Exercise under Physiological and Pathological Conditions, (AME2P), UE3533, Clermont Auvergne University, 63170 Aubiere, France; Camille.CHAMBONNIERE@uca.fr (C.C.); lore.metz@uca.fr (L.M.); david.thivel@uca.fr (D.T.); 2 Auvergne Research Center for Human Nutrition (CRNH), 63000 Clermont-Ferrand, France; mduclos@chu-clermontferrand.fr; 3 National Research Institute for Agriculture, Food and Environment (INRAE), 63000 Clermont-Ferrand, France; 4 Healthy Active Living and Obesity Research Group, Children’s Hospital of Eastern Ontario Research Institute, Ottawa, ON K1H 8L1, Canada; jpchaput@cheo.on.ca; 5 Department of Sport Medicine and Functional Explorations, Clermont-Ferrand University Hospital, G. Montpied Hospital, 63000 Clermont-Ferrand, France; 6 UFR Médecine, Clermont Auvergne University, BP 10448, 63000 Clermont-Ferrand, France

**Keywords:** academic achievements, active desks, adolescent, children, physical activity, sedentary behavior

## Abstract

The purpose of this systematic review was to examine the effects of active desks in the school setting on sedentary behavior, physical activity, academic achievements and overall health among children and adolescents aged 5–17 years. A systematic literature search was conducted using five databases until October 2020. Twenty-three studies were included. Studies reported an increase of around 36% in energy expenditure for cycling desks and between 15% and 27.7% for upright active desks. Children increased inhibitory control and selective attention capacity while using cycling desks. A heterogeneous quality of design and of results were observed limiting comparisons and conclusions for each active desk. Despite the lack of strong methodology for the included studies, active desks appear to be a promising intervention in classrooms to improve health-related outcomes in children aged 5–17 years. Due to weak methodology, future studies with stronger study designs and methodology are needed to better inform policy and practice about the role of classroom active desks on health-related outcomes in children and adolescents.

## 1. Introduction

Concerns and research regarding the effects of sedentary behaviors and physical inactivity on overall health have been growing for the last decades, leading nowadays to a better identification of their independent and joint implications [1,2]. While sedentary behaviors is defined as any waking behavior characterized by an energy expenditure ≤1.5 metabolic equivalents, while in a sitting, reclining or lying posture [3,4], physical inactivity is typically defined as “the non-achievement of physical activity guidelines” [5]. Both sedentary behaviors and physical inactivity have substantially increased in our societies, with physical inactivity being identified as the main cause for about 1.6 million deaths worldwide [6] and leading to a public health cost of $53.8 billion per year [7]. Due to their important implication in the risks of all-cause mortality and cardio-metabolic morbidity as well as in some cancer occurrence [8], both sedentary behaviors [9,10] and physical inactivity [11,12] are of public health concern today.

In children and adolescents, it has been found with device-based measurements that daily sitting time takes over 50% of the waking day at 7 years and 75% at 15 years [13]. This high level of sedentariness, combined with the fact that about 80% of children and adolescents are inactive (i.e., not reaching the physical activity recommendations) [14,15], led some scientists to propose the existence of what they called a “Sedentary & Inactive” profile [16]. Not only physical inactivity and sedentary behaviors have been found to be associated with early metabolic and cardiovascular risk in children and adolescents [17,18,19,20,21], they have also been found to be related to a decrease in cognitive performance and academic achievements [22,23,24].

Knowledge and behaviors developed during childhood have been shown to influence their future behaviors as adults [25]. In particular, children’s physical activity and sedentary behaviors have been shown to not only determine their actual health but also their adolescent and adult behaviors and health [26]. Since children spend at least one third of their waking time in class [27], school appears as an ideal setting to promote health and induce behavioral change [28]. Targeting school time and the school place to promote healthy active behaviors necessitates however to face the highly sedentary nature of the children’s class time. In that context, the literature shows a growing number of experiments trying to implement interventions aimed at breaking and reducing this sedentary time during class [29,30]. The use of active desks in the classroom (e.g., standing desks, sit-to-stand desks, cycling desks, stability balls) has been especially studied [31,32,33,34], with studies showing for instance that sit-to-stand desks seem to reduce sedentary time in the classroom [31] or increase energy expenditure with the use of bike desks [34]. These studies are providing some promising results and our aim is to conduct a systematic analysis of these works to have a better understanding of their effects.

Previous reviews have examined the effects of standing desks on children and adolescents [35,36]. Regarding, active desks, while some already systematically reviewed their effects on academic achievement [37] or questioned their use among specific groups (e.g., overweight and obese) [38], no review has specifically studied the impact of classroom active desks on cognitive, academic and overall health-related (physical, metabolic and mental health) outcomes among children and adolescents. Having a global picture on the role of classroom active desks on improving health-related outcomes of children and adolescents is needed to inform policy and practice.

Thus, the objective of the present systematic review was to analyze the existing literature on the implementation of active desk in the school environment and examine their effects on physical activity, sedentary behavior, academic achievements and overall health in children and adolescents aged 5–17 years.

## 2. Methods

This research is registered in PROSPERO as CRD42020196096. This review was completed in accordance with the Preferred Reporting Items for Systematic Reviews and Meta-Analyses (PRISMA) to identify and collate studies [39].

### 2.1. Search Strategy

A literature search was conducted for studies from year 1990 to October 2020 using the following electronic bibliographic databases: PubMed, ScienceDirect, ResearchGate, Google Scholar and Medline (Cochrane Library). The search terms included the key words “desk* or workstation* or work station” AND “treadmill OR pedaling OR cycling OR bicycl* OR bik* OR active OR exercise ball* OR swiss ball* OR stability ball* OR dynamic seating OR active sitting OR standing OR stepping OR stand up OR position, standing OR standing position* OR sit-to-stand OR sit stand OR stand/sit OR stand biased OR adjustable furniture OR height adjustable” AND “school* OR class* OR child* OR student* OR academic institution”. To identify articles potentially missed during the literature search, reference lists of candidate articles were reviewed.

### 2.2. Eligibility Criteria

#### 2.2.1. Inclusion Criteria

Our selection criteria were specified in advance and included the following: published in English peer-reviewed journal and available in full text [1]; randomized controlled design, non-randomized controlled design, non-randomized design, randomized design, cross-over design [2]; included children and adolescents aged 5–17 years, normal-weight, overweight or obese [3]; included active desks in comparison with traditional desks or within-subject [4]; and experiments were conducted in the school classroom [5]. The following outcomes were reported if they were assessed at least at baseline and follow-up: body composition, sedentary behaviors and physical activity, energy expenditure, cognitive and academic performance, fatigue and musculoskeletal pain symptoms, process evaluation, cardiometabolic health and physical fitness. All intervention durations were included.

#### 2.2.2. Exclusion Criteria

Studies were excluded for: (1) non-school-aged participants or mixed groups (e.g., school-aged children and adults); (2) study with mixed intervention (e.g., active desks coupled with active breaks); (3) study where participants already experimented active desks before the study; (4) specific population with health issues; and (5) studies where authors did not reply to our requests for more complete data or full-text.

### 2.3. Synthesis of Results

Authors have collectively elaborated the structure of the tables based on the different active desks (as described in Table 1). Table 2 presents the included studies as follows: article, study design, school description, sample description, type of active desk, intervention description and outcomes. Table 3 and Table 4 report the different outcomes studied.

**Table 1:** Active desks characteristics and range of price.

Active Desk Type	Description	Range of Price (USD)	Pictures
Upright active desk	Corresponds to standing desk, sit-to-stand desk or stand-biased desk.	150–900	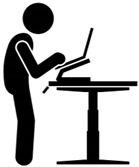 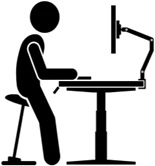
Cycling desk	Is a stationary bike with a desk enabling individuals to work while cycling	200–900	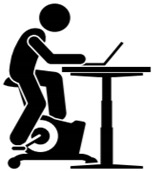
Stability ball	Replace the traditional chair with a stability ball on individual desk	10–100	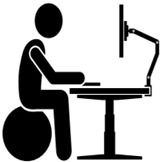

**Table 2:** Participants’ characteristics of included studies for the systematic review.

Author (Country)	Study Design	School Description	Sample Description	Type of Active Desk	Intervention Description	Outcomes
Upright active desk						
Benden et al. (2011) (Texas, USA) [40]	RCT *	1 E	N = 58 Age: NR Sex: NR Grade: 1 BMI: NR Race/ethnie: NR Total study groups: 4 classrooms (2 IG and 2 CG) IG (N = 31) CG (N = 27)	Upright active desks + stools	Treatment length: 9 months (2009–2010 school year) Active desks: individuals Active desks usage time per week: all the school day	EE Body composition (body mass, BMI, body fat percentage) Method: BodyBugg Armband
Benden et al. (2012) (Texas, USA) [41]	NRT	1 E	N = 9 Age range: 6–8 y Grade: 1st grade Sex: girls 33% BMI: 19.5 ± 4.3 kg·m^2^ Race/ethnie: NR	Upright active desks + stools	Treatment length: 5 months Active desks: individuals They used traditional desk on fall semester before swapping stand-biased for spring semester Active desks usage time per week: own volition	Body composition (weight, BMI) EE Step count Methods: Bodybugg Armband A digital scale
Benden et al. (2014) (Texas, USA) [42]	RCT	1 E	N = 326 Mean age: 8.5 y Sex: girls 51.23% Grade: 2, 3, 4 Race/ethnie: White (70.55%), Black (10.12%), Hispanic (10.74%), Asian (7.98%), Native American (0.61%) Total study groups: 8 classrooms (4 IG and 4 CG) Interventional group (N = 202) BMI IG: 17.44 ± 3.26 kg·m^2^ Overweight and obesity IG: 16% overweight and 13% obesity Control group (N = 124) BMI CG: 17.73 ± 3 kg·m^2^ Overweight and obesity CG: 5% with overweight and 16% with obesity	Upright active desks + stools	Treatment length: 9 months (2012–2013 school year) Active desks: individuals Active desks usage time per week: all the school day	Body composition (BMI, body mass) Step count EE Method: Sensewear armband
Blake et al. (2012) (Texas, USA) [43]	NRCT *	1 E	N = NR Age range: 6–7 y Grade: 1^st^ Sex: NR BMI: NR Race/ethnie: NR Total study groups: 5 groups (2 IG, 2 CG, 1 within-group comparisons)	Upright active desks + stools	Treatment length: 1 year Active desks: individuals Active desks usage per week: own volition	Sitting and standing time EE On-task behavior (concentration, engagement) Process evaluation Methods: BodyBugg armband Interviews
Clemes et al. (2016) (Melbourne, Australia **) [31]	RCT *	1 E	N = 44 Mean age: 11.6 ± 0.5 y Grade: 6 Sex: girls 56.8% BMI: 19.4 ± 3.3 kg·m^2^ Race/ethnie: NR Total study groups: 2 classrooms (1IG and 1CG) IG (N = 24) CG (N = 20)	Upright active desks	Treatment length: 10 weeks (September–Novembrer 2013) Active desks: individuals Active desks usage time per week: children were encouraged to stand at least one 30-min class per day	Sitting, standing and stepping time Step counts Method: Accelerometers (activPAL)
Clemes et al. (2016) (Bradford, England **) [31]	NRCT *	E	N = 54 Mean age: 10.0 + 0.3 y Age range: 9–10 y Grade: 5 Sex: NR BMI: 18.3 + 3.2 kg/m2 Race/ethnie: NR Total study groups: IG (N = 27) CG (N = 27)	Upright active desks	Treatment length: 9 weeks (January–April 2014) Active desks: 6 sit-to-stand desks Active desks usage time per weeks: once a day for at least 1 h	Sitting, standing and stepping time Step counts Method: Accelerometers (activPAL)
Clemes et al. (2020) (Bradford, England) [44]	RCT *	8 E	N = 176 Mean age: 9.3 ± 0.5 y Age range: 9–10 y Sex: girls 44.3% Grade: 4–5 Race/ethnie: White British (35.8%), South Asian (48.3%), Other (15.9%) Total study groups: 8 classrooms (4 IG and 4 CG) Interventional group (N = 86) mean age: 9.3 ± 0.4 y BMI: 18.2 ± 3.3 kg·m^2^ Control group (N = 90) mean age: 9.3 ± 0.5 y BMI: 18.2 ± 4.0 kg·m^2^	Upright active desks + stools	Treatment length: 4–5 months (February-July 2017) Active desks: 6 active desks replaced 3 standard desks in IG Active desks usage time per week: at least 1 h/day	Process evaluation PA (MVPA, LPA) Sitting, standing and stepping time Blood pressure Body composition (BMI, body mass) On-task behavior (concentration, engagement) Musculoskeletal discomfort Methods: Inclinometer (ActivPAL3) Bio-impedance (Tanita DC-360S) Accelerometer (ActiGraph GT3x + ) Interview, focus groups Questionnaires Semi-automated recorder (Omron HEM-907)
Dornhecker et al. (2015) [45]	NRCT	3 E	N = 282 Age range: 7–10 y Grade: 2, 3, 4 Sex: girls 53.18% BMI: NR Race/ethnie: Black (12.68%), Hispanic (10.49%), Asian (7.62%), White (69.22%) Total study groups: 2 groups (1 IG and 1 CG) Interventional group (N = 158) Grade 2 (35.44%), Grade 3 (45.57%), Grade 4 (18.99%) Control group (N = 124) Grade 2 (43.55%), Grade 3 (33.87%), Grade 4 (22.58%)	Upright active desks + stools	Treatment length: 5 months Active desks: NR Active desks usage per week: NR	On-task behavior (concentration, engagement) Method: Behavioral Observations of Students in Schools (BOSS)
Ee et al. (2018) (Perth, Australia) [32]	Cross-over	1 E	N = 47 Age range: 10–11 y Grade: 4 Sex: boys 100% BMI: NR Race/ethnie: NR	Upright active desks	Treatment length: Academic year (2016–2017) Active desks: individuals They used 21 school day a standing desk, then they swapped during 21 school day with traditional desk. This swapping continued throughout the school year Active desks usage time per week: all the school day.	Standing and sitting time Sedentary time PA (MVPA, LPA) Musculoskeletal discomfort Methods: Accelerometers (ActiGraph GT9X Link) Nordic Musculoskeletal Questionnaire
Kidokoro et al. (2019 ) (Nagano, Japan) [46]	NRCT	1E	N = 38 Mean age: 11.3 ± 0.5 y Age range: 11–12 y Grade: 6 Sex: girls 42% BMI: IG: 18.3 ± 3.1 CG: 17.4 ± 3.3 Race/ethnie: NR Total study groups: 2 groups (1 IG and 1 CG) Interventional group (N = 18) Mean age: 11.3 ± 0.5 y BMI: 18.3 ± 3 kg·m^2^ Control group (N = 2O) Mean age: 11.3 ± 0.5 y BMI: 17.4 ± 3.3 kg·m^2^	Upright active desk	Treatment length: 6 months (July–December 2018) Active desks: individuals Active desks usage per week: own volition	PA (LPA, MVPA) Sedentary time Process evaluation Methods: Accelerometers (ActiGraph) Questionnaire (Likert-type scale)
Koepp et al. (2012) (Idaho, USA) [47]	NRT	1 E	N = 8 Mean Age: 11.3 ± 0.5 y Grade: 6 Sex: girls 37.5% BMI: 19.4 ± 5.3 kg·m^2^ Race/ethnie: NR	Upright active desk + stools	Treatment length: 5 months (January–June 2010) Active desks: individuals Active desk usage time week: own volition	Step count Body composition (BMI, weight) Musculoskeletal discomfort On-task behavior (concentration, engagement) Methods: Podometers Observations
Mehta et al. (2015) (Texas, USA) [48]	NRT *	1 S	N = 27 Mean age: 14.30 ± 0.61 y Grade: NR Sex: girls 70.6% BMI: 23.27 ± 4.44 kg·m^2^ Race/ethnie: White (41%), Hispanic (52%), Black (4%), Asian (4%)	Upright active desks	Treatment length: academic year, 27 weeks of continued exposure Active desks: individuals Active desks usage time per week: own volition	Neurocognitive function Prefrontal Cortex (PFC) Activity Methods: The Psychology Experiment Building Language Wisconsin Card Sorting Task (WCST) Flanker Task (FT) Memory Span Task (MST) Trail-Making Task (TMT) Stroop Color Word Task (SCWT) Functional near infrared spectroscopy (fNIRS)
Parry et al. (2019) (Perth, Western Australia) [49]	Cross-over	E	N = 23 Age range: 9–11 y Grade: 4 Sex: boys 100% BMI: NR Race/ethnie: NR	Upright active desk	Treatment length: academic year (2017) Active desks: individuals They used 21 school day a standing desk, then they swapped during 21 school day with traditional desk. This swapping continued throughout the school year Active desks usage time per week: all the school day	Sitting and standing time Sedentary time and PA (MVPA, LPA) Musculoskeletal discomfort Methods: Accelerometers (Actigraph GT9X Link) Modified version of the Nordic Musculoskeletal Questionnaire Focus groups, interview
Pickens et al. (2016) (Texas, USA) [50]	RT	1 S	N = 18 Age: NR Grade: NR Sex: girls 72% BMI: NR Race/ethnie: Hispanic (66%), White (30%)	Upright active desks + stools	Treatment length: 3 months Active desks: individuals Active desks usage time per week: NR	Step count Sitting, standing time Method: Inclinometer (ActivPal3™)
Sherry et al. (2020) (Bradford, UK) [51]	CT *	2 E	N = 49 Age range: 9–10 y Grade: 5 Sex: 53.8% BMI: IG: underweight (9.1%), normal (63.6%), overweight (13.6%), obese (18.2%) CG: underweight (3.9%), normal (61.5%), overweight (11.5%), obese (23.1) Race/ethnie: South Asian (69.4%), White British (26.5%), Other (4%) Total study groups: 2 groups (1 IG and 1 CG) IG (N = 27) CG (N = 22)	Upright active desks + stools	Treatment length: 8 months (November 2015 to July 2016) Active desks: individuals Active desks usage time per week: at least 20 min per classroom day	Musculoskeletal discomfort Cognitive function Process evaluation Sitting, standing, stepping time Methods: Inclinometer (ActivPAL) Questionnaires Stroop test Corsi Block Tapping test Interviews, Observations
Sprengeler et al. (2020) (Ludwigsburg, Germany) [52]	Cross-over	1 E	N = 52 Mean age: 8.4 ± 0.7 y Age range: 8–10 y Grade: 3 Sex: 61.5% BMI: normal weight (78.9%), overweight/obese (21.1%) Race/ethnie: NR	Upright active desks + stools	Treatment length: 3 months (January-March 2018) Active desks: individuals. 32 desks were equally distributed among the three classes Active desks usage time per week: the group 1 used active desks during 3 weeks (February, T1) and after were assigned to the traditional working desks until March (T2). The group 2 begin to used traditional desks until February (T1) and after used active desks (T2). A washout period of 2 weeks is present between T1 and T2	Standing, and sitting time Methods: Inclinometer (ActivPAL)
Sudholz et al. (2016) (Melbourne, Australia) [53]	NRCT *	1 S	N = 41 Mean age: 13.7 ± 1.4 y Age range: 12–16 y Grade: 7, 9, 10 Sex: girls 49% BMI: NR Race/ethnie: NR Total study groups: 2 groups (1 IG and 1 CG) IG (N = 27) CG (N = 14)	Upright active desks + stools	Treatment length: 7 weeks (August to October 2014) Active desks: individuals Active desks usage time per week: own volition	Sitting and standing time/bouts Sedentary time PA (LPA) Feasibility/process evaluation Musculoskeletal discomfort Methods: Accelerometer (ActiGraph3X) Inclinometer (ActivPAL3C) Questionnaire
Swartz et al. (2019) (USA) [54]	Cross-over	1 E	N = 99 Mean age: 10.2 ± 1.4 y Grade: 3, 4, 6 Grade 3 (N = 22) Grade 4 (N = 36) Grade 6 (N = 41) Sex: girls 42.9% BMI: Grade 3: 55th BMI percentile Grade 4: 43rd BMI percentile Grade 6: 61st BMI percentile Race/ethnie: White (69%), black (3%), Asian (8%), mixed race (7%), Hispanic (8%)	Upright active desks + stools	Treatment length: 9 weeks Active desks: individuals. Half of the students used a stand-biased desk and half used a sitting desk. The Stand-Sit group used a stand-biased desk for 9 weeks (September to December) and sitting desk for 9 weeks (January to April). The Sit -Stand group used a sitting desk for 9 weeks (September to December) and stand-biased for 9 weeks (January to April). Active desks usage time per week: NR	Sedentary time PA (LPA, MVPA) Method: Accelerometer (ActigraphGT3X+ or wGT3X-BT)
Verloigne et al. (2018) (Flanders, Belgium) [55]	RCT	10 E and 9 S	N = 343 Age range: 10–16 y Grade: 5, 10 Sex: girls 54.5% BMI: NR Race/ethnie: NR Total study groups: IG: 5 primary, 5 secondary CG: 5 primary 4 secondary	Upright active desks	Treatment length: 6 months (January–June 2017) Active desks: 3 standing desks were placed in each intervention class Recommendations of active desks usage time per week: rotations every half lesson hour (25 mn)	Process evaluation Sitting, standing and stepping time Methods: Inclinometer (ActivPAL) Focus groups (children) and interviews (teachers) The Paediatric Quality of Life Inventory (PEDS-QL) EuroQol 5dimension Youth (EQ-5D-Y
Wendel et al. (2016) (Texas, USA) [56]	RCT	3 E	N = 193 Mean age: 8.8 y Grade: 3, 4 Sex: girls 50.3% BMI: normal (79.3%), overweight (11.9%), obese (8.8%) Race/ethnie: White (74.6%), Asian (10.4%), Hispanic (7.8%), Afro-American (7.3%) Total study groups: 4 groups (IG, CG, CG-IG, IG-CG) IG (N = 62) CG (N = 49) IG-CG (N = 59) CG-IG (N = 23)	Upright active desks + stools	Treatment length: 2 years (2011–2013) Active desks: individuals Active desks usage time per week: NR	Body composition (BMI)
Wick et al. (2018) (Swiss) [57]	NRCT *	2 E	N = 38 Age range: 10–12 y Grade: NR Sex: girls 42% Race/ethnie: NR Total study group: 2 groups (1 IG and 1 CG) Interventional group (N = 19) Mean age: 10.8 ± 0.6 y BMI: 18.0 ± 2.8 kg·m^2^ Control group (N = 19) Mean age: 10.8 ± 0.8 BMI: 18.8 ± 4.3 kg·m^2^	Upright active desks	Treatment length: 11 weeks (August–December 2014) Active desks: individuals Active desks usage per week: teachers encouraged students to work for about 60 min a day at the active desk	Sitting, standing and walking time Cognitive function Methods: Accelerometers (ActiGraph) Observations Self-reporting Digit span task test Eriksen flanker test
Cycling desk						
Fedewa et al. (2017) (South-eastern region, USA) [30]	RCT *	1 S	N = 17 Age range: 14–18 y Grade: 8–12 Sex: NR BMI: NR Race/ethnie: NR Total study groups: 6 classrooms (3 IG and 3 CG) IG (N = 11) CG (N = 6)	Bike desks	Treatment length: academic year Actives desks: 4 FitDesks per IG classroom. Actives desks usage time per week: access to FitDesks for the entire day with the exception of lunch, and extracurricular courses including physical education, computer lab, and art.	Sedentary time PA (MVPA, LPA, vector magnitude) Process evaluation EE Methods: Accelerometers (ActiGraph GT3X) Interviews Questionnaire
Ruiter et al. (2019) (Germany) [58]	NRT	2 E	N = 38 Age: 12.50 ± 0.62 y Grade: 8 Sex: girls 57% BMI: NR Race/ethnie: NR	Bike desks	Treatment length: 2 weeks Active desks: individuals. Active desks usage time per week: they used bike desks only for the cognitive function’s assessment. All 2 sessions (week 1, week 2) occurred at the same time on separate days with a 7-day interval between tests.	Cognitive capacities Methods: Eriksen Flanker Task Digit Span Task Visual pattern Task Questionnaire
Torbeyns et al. (2017) (Ninove, Belgium) [34]	RCT	1 S	N = 44 Mean age: 14.3 ± 0.6 y Grade: 9–10 Sex: girls 34% Race/ethnie: NR Total study groups: 2 classroom (1 IG and 1 CG) Interventional group (N = 21) BMI: 19.7 ± 3.5 kg·m^2^ Control group (N = 23) BMI CG: 20.1 ± 3.7 kg·m^2^	Bike desks	Treatment length: 22 weeks, 5 months (October-February 2015) Actives desks: individuals; students adjust the cycling intensity to their preference Actives desks usage time per week: IG used a bike desk for 4 class hours/week	PA Body composition (BMI, body mass, body fat percentage, waist circumference) EE Physical fitness Cognitive capacities On-task behavior (concentration, engagement) Methods: SenseWear mini armband The Dutch (Native speech of the participants) version of the Rey Auditory Verbal Learning Test (RAVLT) The Stroop test The Rosvold Continuous Performance Test (RCPT) Continuous electroencephalography (EEG) LOSO attention questionnaire ‘Dutch’ and mathematics test
Stability ball						
Erwin et al. (2016) (USA) [33]	RCT	1 E	N = 43 Age: NR Grade: 4 Sex: girls 32.6% BMI: NR Race/ethnie: White (76%), African American (11%), Asian (7%), Hispanic (3%), other (3%) Total study groups: 2 classrooms (1 IG and 1 CG) IG (N = 23) CG (N = 21)	Stability balls	Treatment length: 12 weeks Active desks: individuals Active desks usage time per week: all the school day	On-task behavior (concentration, engagement) PA (step count, horizontal et vertical accelerometers count) Methods: Accelerometer (ActiGraph) Momentary Time Sampling (MTS)
Fedewa et al. (2015) (USA) [59]	RCT	1 E	N = 67 Age: NR Grade: 2 Sex: girls 48% BMI: NR Race/ethnie: NR Total study groups: 4 classrooms (2 IG and 2 CG) IG (N = 36) CG (N = 31)	Stability balls	Treatment length: 9 months Active desks: individuals Active desks usage time per week: all the school day	Academic performance Discipline referral levels On-task behavior (concentration, engagement) Methods: Measures of Academic Progress “clip downs” (a school wide disciplinary system) Momentary Time Sampling (MTS)

NR: Not Reported; USA: United States of America; RCT: Randomized Controlled Trial; RT: Randomized Trial; NRCT: Non-Randomized Controlled Trial; NRT: Non-Randomized Trial; *: Pilot Study; ** Studies in the same published article; E: Elementary; S: Secondary; IG: Intervention Group; CG: Control Group; CG-IG: switch from a control to an intervention condition; IG-CG: switch from a intervention to a control condition; EE: Energy Expenditure; PA: Physical Activity; BMI: Body Mass Index; LPA: Light Physical Activity; MVPA: Moderate-to-Vigorous Physical Activity.

**Table 3:** Results of body composition, sedentary behaviors, physical activity, energy expenditure, physical capacity and cardiometabolic health in the included studies for the systematic review.

Author (Year)	Body Composition	Sedentary Behaviors and Physical Activity	Energy Expenditure	Physical Capacity and Cardiometabolic Health
Upright active desk				
Benden et al. (2011) [40]	NR	N/A	EE: IG > CG: +0.182 ± 0.080 kcal·min^−1^ (*p* = 0.022) Students in the intervention group IG burned 17% more calories than did those in the control group Overweight/obese EE: IG > CG (IG: 1.56 kcal·min^−1^; CG: 1.18 kcal·min^−1^)	N/A
Benden et al. (2012) [41]	Weight: fall < spring fall vs. spring: fall: 27 ± 7.9 kg vs. spring: 29.5 ± 8.9 kg BMI: fall < spring fall vs. spring: fall: 19.5 ± 4.3 kg·m^2^ vs. spring: 19.8 ± 4.3 kg·m^2^	Steps: Steps within-subjects spring > fall: +17.6% Mean number of steps spring > fall: +836 steps	Spring: >fall: +25.7%: +0.29 kcal·min^−1^ (*p* < 0.0001) Day-to-day variation: Lower EE day 3 and 4 (*p* < 0.0001)	N/A
Benden et al. (2014) [42]	BMI: no significant	Step counts: Fall semester: IG > CG: +1.61 steps/min (*p* = 0.0002) Spring semester: IG > CG (+0.12 steps/min): no significant (*p* = 0.8193) Normal vs. overweight and obese: overweight: 0.78 steps/min (*p* < 0.001); obese: 0.62 steps/min (*p* = 0.0059)	EE: Fall semester: increase IG > CG: +0.16 kcal·min^−1^ (*p* < 0.001) Spring semester: increase IG > CG: +0.08 kcal·min^−1^ (*p* = 0.0092) Normal vs. overweight and obese: overweight: 0.24 kcal·min^−1^ (*p* < 0.001); obese: 0.40 kcal·min^−1^ (*p* < 0.001)	N/A
Blake et al. (2012) [43]	N/A	Standing and sitting time: IG: 66% standing at their desks as opposed to sitting	EE: IG > CG: +17% burned calories	N/A
Clemes et al. (2016) (Melbourne, Australia) [31]	N/A	During class: Sitting time: IG pre > post: pre vs. post: pre: 67.9 ± 8.4% vs. post: 58.5 ± 8.4% (*p* < 0.001) Standing time: IG pre < post pre vs. post: pre: 18.1 ± 4.5 vs. post: 26.4 ± 7.5% (*p* < 0.001) Stepping time: no significant Step counts: no significant Sitting time (%) IG < CG (*p* = 0.03) Standing time (%) IG > CG (*p* < 0.01) Whole weekday: Standing time IG pre < post pre vs. post: pre: 21.3 ± 6.1% vs. post: 25.5 ± 5.5% (*p* < 0.01) Sitting time: no significant Stepping time: no significant	N/A	N/A
Clemes et al. (2016) (Bradford, England*) [31]	N/A	During class: Sitting time IG pre > post: IG pre vs. post: pre: 71.8 ± 10.6% vs. post 62 ± 15.8% (*p* = 0.03) Standing time: no significant Stepping time IG pre < post IG pre vs. post: pre: 8.2 ± 2.8% vs. post: 14.5 ± 7.9% (*p* = 0.002) Step counts IG pre < post IG pre vs. post: pre: 1654 ± 528.9 vs. post: 3024 ± 2195 (*p* = 0.013) Difference IG and CG: no significant Whole weekday: Sitting, standing and stepping time: no significant	N/A	N/A
Clemes et al. (2020) [44]	Body mass: IG < CG IG vs. CG: IG: 37.7 ± 8.7 kg vs. CG: 39.2 ± 10.6 kg Percent body fat: Girls: IG > CG IG vs. CG: IG: 25 ± 8.3% vs. CG: 23.7 ± 9.1% Boys: IG < CG IG vs. CG: IG: 19 ± 6.6%; CG: 20.7 ± 8.9% BMI: IG > CG IG vs. CG: IG: 18.8 ± 3.5 kg·m^2^ vs. CG: 18.7 ± 4.1 kg/m²	↓ Sitting time IG (−30.6 min/day) Sitting time: IG < CG IG vs. CG: IG: 472 ± 73.5 min/day vs. CG: 504.4 ± 94 min/day Standing time: IG > CG IG vs. CG: IG: 197.1 ± 49.4 min/day vs. CG: 176.5 ± 45.7 min/day Stepping time: IG > CG IG vs. CG: IG: 166.4 ± 41.9 min/day vs. CG: 150 ± 42.1 min/day LPA: IG post > pre post vs. pre: post: 392.7 ± 70.8 min/day vs. pre: 383.5 ± 68.6 min/day MVPA: IG post > pre post vs. pre: post: 45.7 ± 24.7 min/day vs. pre: 37.4 ± 17.9 min/day	N/A	Systolic blood pressure: IG > CG IG vs. CG: IG: 110.5 ± 11.2 mmHg vs. CG: 107.3 ± 11.7 mmHg Diastolic blood pressure: IG > CG IG vs. CG: IG: 68.4 ± 9.7 mmHG vs. CG: 66.3 ± 9.5 mmHg
Ee et al. (2018) [32]	N/A	School standing and sitting: IG and CG: sitting time (61%) > standing time (19%) Standing time: IG > CG IG vs. CG: IG: 84 ± 4 min/day vs. CG: 63 ± 3 min/day (*p* < 0.001) Sitting time: IG < CG IG vs. CG: IG: 208 ± 6 min/day vs. CG: 231 ± 5 min/day (*p* = 0.003) Whole Day Physical Activity and Sedentary Time Sedentary time: no significant: IG vs. CG: IG: 674 ± 23 min/day vs. CG: 686 ± 26 min/day LPA: no significant IG vs. CG: IG: 241 ± 7 min/day vs. CG: 256 ± 6 min/day MVPA: no significant Moderate: IG vs. CG: IG: 39 ± 2 min/day vs. CG: 42 ± 2 min/day Vigorous: IG vs. CG: IG: 18 ± 1 min/day vs. CG: 20 ± 1 min/day	N/A	N/A
Kidokoro et al. (2019) [46]	N/A	During classroom Sedentary behaviors: IG < CG IG vs. CG: IG: 59% vs. CG: 67% (*p* = 0.035) SB: IG post < pre: −18.3 min/day LPA: no significant MVPA: IG > CG IG vs. CG: IG: 12.5% vs. CG: 8.3% (*p* = 0.005) MVPA: IG pre < post: +19.9 min/day	N/A	N/A
Koepp et al. (2012) [47]	Weight: pre < post: pre vs. post: pre: 41.4 kg vs. 44.5 kg (*p* = 0.0007) BMI: pre and post: no significant pre vs. post: pre: 19.4 ± 5.3 kg/m² vs. post: 19.3 ± 5.2 kg.m^2^ (*p* < 0.3416)	Step counts: no significant pre vs. post: pre 1886 ± 809 steps vs. post: 2248 ± 990 steps (*p* > 0.1127)	N/A	N/A
Parry et al. (2019) [49]	N/A	For IG vs. CG: Standing time: > at the start of the school year (+17 min/school day) and at the end (+26 min/school day) Sitting time: <at the start of the school year (−17 min/school days) and at the end (−40 min/school day) Standing and sitting time over time: IG and CG: no significant (*p* = 0.062) Physical activity and sedentary time: no significant	N/A	N/A
Pickens et al. (2016) [50]	N/A	Sitting time: pre > post pre vs. post: pre: 1032.4 min vs. post: 857.6 min (*p* < 0.0001) Standing time: pre < post pre vs. post: pre: 203.7 min vs. post: 353 min (*p* < 0.001) Steps: pre < post pre vs. post: pre: 6611.6 vs. post: 8898.4 (*p* = 0.0619)	N/A	N/A
Sherry et al. (2020) [51]	N/A	During class time Sitting time: IG < CG IG vs. CG: IG: 52.4 ± 21.9% vs. CG: 72.1 ± 6.6% (*p* = 0.001) Standing time: IG > CG IG vs. CG: IG: 35.6 ± 18.1% vs. CG: 17.6 ± 9.0% (*p* = 0.001) Stepping time: IG > CG IG vs. CG: IG: 12.0 ± 4.0% vs. CG: 11.0 ± 2.7% (*p* = 0.035) Sit-to-stand transitions: IG > CG IG vs. CG: IG: 10.7 ± 2.3 p/h vs. CG: 5.6 ± 2.2 p/h (*p* < 0.001) Behavior after school: no significant Full weekday Sitting time: IG < CG IG vs. CG: IG: 59.1 ± 10.3% vs. CG: 63.5 ± 9.7% (*p* = 0.042)	N/A	N/A
Sprengeler et al. (2020) [52]	N/A	During lessons: Sitting time: G1: pre > mid: −13.1% G2: pre > mid: −9.78% Standing time: G1: pre < mid: 11.6% G2: pre < mid: 8.63% During school breaks: Sitting: G1: pre > mid: −10.3%, pre > post: −11.8% G2: pre > mid: −11.8%, pre > post: −8.59% Standing: G1: pre < mid: 6.20% G2: pre < mid: 7.82%, pre < post: 8.08%	N/A	N/A
Swartz et al. (2019) [54]	N/A	During classroom Sedentary and active time post: IG + CG: ↑ sedentary time (*p* < 0.001) ↓ active (*p* < 0.001) Sedentary behavior: IG pre-post (+2.4%) < CG pre-post (+6.5%) (*p* = 0.038) LPA: time I: no significant (*p* = 0.314) MVPA: during classrooms IG pre-post (−0.7%) < CG pre-post (−5.0%) (*p* = 0.001) Predictive outcomes Sedentary behavior: students with high sedentary time at baseline have more finals effects (*p* = 0.029) LPA: IG and CG: no significant (*p* = 0.773) MVPA: students with high sedentary time at baseline have more finals effects (*p* < 0.0001)	N/A	N/A
Verloigne et al. (2018) [55]	N/A	(Questionnaire data) Primary school Self-efficacy to break up sitting time: IG pre < mid pre vs. mid: 3.2 ± 0.2 vs. 3.4 ± 0.2 (beta = 0.188) Habit of breaking up sitting time: CG pre < post pre vs. post: 3.6 ± 0.2 vs. 3.4 ± 0.2 (beta = 0.467) Secondary school: ↓ Sitting time: IG < CG pre-mid test, beta = 0.058 IG pre vs. mid: 275.8 ± 11.4 min/day vs. 366.5 ± 11.4 min/day CG pre vs. mid: 362.2 ± 12.5 min/day vs. 314.7 ± 12.5 min/day Breaking up sitting time: IG pre > mid pre vs. mid: 3.7 ± 0.1 vs. 3.5 ± 0.1 (beta = −0.456) (ActivPAL) Primary school Sitting time: during school hours: IG pre > mid pre vs. mid: 243.8 ± 8.9 min vs. 217.9 ± 8.9 min (beta = −37.404) Standing time: during schools hours/entire school day: IG pre < post school hours: pre vs. mid: 105.6 ± 7.5 min vs. 131.2 ± 7.5 min, beta = 34.148 entire school day: pre vs. mid: 195.1 ± 11.1 min vs. 220.5 ± 11.1 min (beta = 34.464) Stepping time: during entire school day: IG pre > mid: −7 min pre vs. mid: 141.5 ± 8.3 min vs. 134.1 ± 8.3 min (beta = −18.796) Sitting time: during school hours: IG ↓ (−26 min) vs. CG ↑ (+12 min) Time spent in sitting bouts during school hours: IG ↓ (−19 min) vs. CG ↑ (+11 min) Time spent in sitting bouts across the whole school day: IG ↓ (−27 min) vs. CG ↑ (+18 min) Standing time: during school hours: ↑ IG (+26 min) vs. CG ↓ (~10 min) Time spent in standing bouts during school hours: ↑ IG (+29 min) vs. CG ↓ (~10 min) Time spent in standing bouts across the whole school day: ↑ IG (+25 min) vs. CG ↓ (~10 min) Secondary school: Number of sit-to-stand transition: IG pre > mid pre vs. mid: 24.7 ± 1.8 vs. 21.7 ± 1.9 (beta = −5.034)	N/A	N/A
Wendel et al. (2016) [56]	BMI IG < CG IG vs. CG (*p* = 0.037) BMI IG and CG-IG/IG-CG: no significant	N/A	N/A	N/A
Wick et al. (2018) [57]	N/A	Sitting time: IG < CG IG vs. CG: IG: 172.1 ± 19.7 min vs. CG: 184.9 ± 13.7 min (*p* = 0.03) Standing time: IG > CG IG vs. CG: IG: 60.5 ± 15.1 min vs. CG: 47.1 ± 11.6 min (*p* = 0.0004) Walking time: no significant IG vs. CG: IG: 19.9 ± 6.3 min vs. CG: 18.9 ± 4.4 min (*p* = 0.57)	N/A	N/A
Cycling desk				
Fedewa et al. (2017) [30]	N/A	Sedentary time: IG < CG: IG vs. CG time 1: IG: 116.3 ± 53.9 min/day; CG: 58.6 ± 10.8 min/day IG vs. CG time 2: IG: 79.2 ± 52.1 min/day vs. CG: 114.5 ± 18.7 min/day LPA: IG > CG IG vs. CG time 1: IG: 19.4 ± 9.2 min/day vs. CG: 6.7 ± 3.0 min/day IG vs. CG time 3: IG: 24.8 min/day vs. CG: 10.2 ± 6.4 min/day MVPA: IG > CG IG vs. CG time 1: IG: 35.6 ± 19.1 min/day; CG: 15.6 ± 6.8 min/day IG vs. CG time 3: IG: 43.0 min/day vs. CG: 30.6 ± 13.2 min/day Vector magnitude: IG > CG IG vs. CG time 1: IG:152 394 ± 83 288 ct/min; CG: 65 908 ± 34 085 ct/min IG vs. CG time 3: IG: 176 119 ct/min vs. CG: 111 429 ± 46059 ct/min	EE: IG time 3 < time 1 Time 3 vs. time 1: time 3: 94.9 ± 29 kcal vs. time 1: 108.9 ± 32.1 kcal	N/A
Torbeyns et al. (2017) [34]	Fat percentage: IG and CG: pre < post pre vs. post: pre: 18.8 ± 9.9% vs. post: 20.1 ± 9.3% (*p* < 0.001) Waist circumference: IG and CG: pre < post pre vs. post: pre: 66.9 ± 6.6 cm vs. post: 68.0 ± 6.0 cm (*p* = 0.017) Body weight: IG and CG pre < post pre vs. post: pre: 56.5 ± 11.3 kg vs. post: 58.1 ± 9.9 kg (*p* < 0.001) BMI: CG post > pre post vs. pre: pre: 20.1 ± 3.7 kg/m²; post:20.5 ± 3.5 kg/m² (*p* = 0.005) IG post and pre: no significant pre vs. post: pre: 19.7 ± 3.5 kg/m² vs. post: 19.9 ± 3.2 kg/m² (*p* = 0.205) BMI CG and IG at T0 and T1: no significant	Physical activity outside the classroom PA: IG and CG pre > post Pre vs. post: pre: 2.6 ± 0.7 vs. post: 2.4 ± 0.7 (*p* < 0.001)	EE: IG EE access bike desks (+36%) > EE normal hours IG bike desks vs. EE IG normal hours: 128.5 ± 34.7 kcal.h^−1^ vs. 94.6 ± 16.7 kcal.h^−1^ (*p*< 0.001) Class hours in which IG had access to the bike desks: EE IG > CG IG vs. CG: 128.5 ± 34.7 kcal.h^−1^ vs. 100.0 ± 16.2 kcal.h^−1^ (*p* = 0.002)	20 m shuttle run test: performance IG post > pre pre vs. post: pre 6.4 ± 2.5; post: 7.0 ± 2.9 (*p* = 0.021) RPE: post IG < CG IG vs. CG: IG: 5.5 ± 1.3; CG: 6.4 ± 1.3 (*p* = 0.047)
Stability ball				
Erwin et al. (2016) [33]	N/A	Difference IG and CG pre and post (*p* < 0.05) Vertical accelerometer counts: (IG and CG) pre > post pre vs. post, pre: 79.56 ± 46.36; post: 51.26 ± 38.11 (*p* < 0.05) Horizontal accelerometer counts: (IG and CG) pre > post pre vs. post: pre:103.92 ± 95.76; post: 62.45 ± 27.41 (*p* = 0.03) Step counts: (IG and CG) pre > post pre vs. post: pre: 4242.01 ± 2006.16; post: 2975.82 ± 1611.20 (*p* < 0.01) PA: IG and CG pre- and post-test: no significant	N/A	N/A

N/A: Not Applicable; NS: Not Significant; NR: Not Reported; BMI: Body Mass Index: EE: Energy Expenditure; PA: Physical Activity; IG: Intervention Group; CG: Control Group; LPA Light Physical Activity; MVPA: Moderate-to-Vigorous Physical Activity; ↓ decrease of; ↑ increase of.

**Table 4:** Results of cognitive and academic performance, fatigue and musculoskeletal pain symptoms and process evaluation in the included studies for the systematic review.

Author (Year)	Cognitive and Academic Performance	Fatigue and Musculoskeletal Pain Symptoms	Process Evaluation
Upright active desk			
Blake et al. (2012) [43]	Attention and focus: IG > CG	N/A	By the fourth week of the intervention, more than two-thirds of the students stopped using the stool completely Teacher’s perspective: Good acceptability of children Active desks considering as “cool” Parents’ perception: positive impact on child’s behavior at school
Clemes et al. (2020) [44]	Learning engagement and disaffection scores: no differences Total difficulties score: IG < CG IG vs. CG: IG: 7.8 ± 6.6 vs. CG: 6.9 ± 6.0 Disruption to the classroom: no adverse effects	Musculoskeletal discomfort: no adverse effects	Overall recruitment: rate being 33% (95% CI: 16 to 55%) Parental consent: 75% At follow-up, retention of participating children was 97%
Dornhecker et al. (2015) [45]	Academic engagement: Fall semester: IG > CG: +4.21 (*p* = 0.003) Spring semester: IG > CG: +0.72 (*p* = 0.003) Differences 2nd grade and 3rd grade students: no significant (*p* = 0.39) Difference 4th grade and 3rd grade: no significant (*p* = 0.19)	N/A	N/A
Ee et al. (2018) [32]	N/A	Neck discomfort: IG < CG (*p* = 0.005) Other body parts: no significant	N/A
Kidokoro et al. (2019) [46]	N/A	N/A	Children’s perception: Enjoying classes using standing desks: 66.7% Expressed willingness to continue using their standing desks: 72.2% Felt that they could express their thoughts more effectively: 66.7% Found it easier to work: 77.7% Felt less sleepy when using standing desk: 97.8% Felt fatigued in the standing classroom: 11.2% Usage of standing desks: 21.4 ± 5.9 min/day Changing their posture: 1.8 ± 0.8 times during a class
Koepp et al. (2012) [47]	Classroom management: no significant (*p* < 0.5) Concentration: no significant (*p* < 0.81)	Discomfort: no significant (*p* < 0.06)	N/A
Mehta et al. (2015) [48]	Cognitive performance: (executive function and working memory tasks) post: +7–14% Neurocognitive assessments: Wisconsin Card Sort: Reaction time pre vs. post: −10% (*p* < 0.0001) Correct responses pre vs. post: +14% (*p* = 0.014) % Correct responses pre vs. post: +13% (*p* = 0.016) Flanker test: Reaction times for congruent pre vs. post: no significant (*p* = 0.112) Reaction times for incongruent pre vs. post: no significant (*p* = 0.079) Percent correct congruent pre vs. post: no significant (*p* = 0.18) Percent correct incongruent responses pre vs. post: no significant (*p* = 0.749) Memory Span test: pre vs. post: no significant (*p* = 0.09) Trail Making Test: pre vs. post: no significant (*p* > 0.205) TMT letters: −7% (*p* = 0.012) TMT number + letter: −14% (*p* > 0.0001) Stroop Color Word: Reaction times: pre > post: −13% (*p* = 0.001) Percent correct responses: pre vs. post: no significant (*p* = 0.239) Prefrontal cortex Activity: nHbO2 levels across all five tasks: pre vs. post: no significant (*p* > 0.212) Effect of hemisphere: no significant (*p* > 0.194) Time point × hemisphere interactions: Wisconsin Card Sorting Task (*p* = 0.042) Memory Span Task (*p* = 0.05) Trail-Making Task (*p* = 0.033) Stroop Color Word test: nHbT left hemisphere > right hemisphere: 19.22 (*p* = 0.001) Others test: no significant across hemispheres and interaction with time points (*p* > 0.117)	N/A	N/A
Parry et al. (2019) [49]	N/A	Musculoskeletal discomfort: neck and shoulder pre > post Neck: (*p* = 0.004) Shoulder: (*p* < 0.001)	N/A
Sherry et al. (2020) [51]	Cognitive function: Corsi Block Tapping: no significant Stroop test: no significant	Musculoskeletal discomfort: Whole body: no significant Upper limb: no significant Neck and back: no significant Lower limb: no significant	Children’s perception: Like sit-to-stand transition because sitting can become uncomfortable Improve behavior in class: children stay in the same place One child feels the classroom noisier Teacher’s perception: Need lot of place Need to adapt his teaching After the 20-min period standing, 22 out 27 children immediately chose to sit back down.
Sudholz et al. (2016) [53]	N/A	Musculoskeletal discomfort: Got pain in legs or back: while standing during lessons: 51%	Children’s perceptions: Continue to used actives desks: 70% Worked well during lessons: 69% Enjoying lessons more since the actives desks were introduced: 54% Felt more energetic across the day: 46% Concentrated better on doing my work: 44% Was easily distracted: 36% Was too tired: to be active after school: 18% Teachers’ perceptions: Continue teaching with the height adjustable desks: 71% Adolescent standing during lessons: Negatively influenced ability to work effectively: 14% Results in loss of concentration: 14% Increase ability to complete tasks: 29% Were too disruptive: 0%
Verloigne et al. (2018) [55]	N/A	N/A	Primary schools: Frequency of using the standing desks: IG mid > post mid vs. post: 2.94 ± 0.61 times/week vs. 1.80 ± 0.61 times/week (beta = −0.379) Mean duration at the desks: IG mid > post mid vs. post: 84.31 ± 13.03 min.week^−1^ vs. 57.69 ± 13 min.week^−1^ (beta = −0.376) Self-efficacy to use the desk: IG mid > post mid vs. post: 4.12 ± 0.15 vs. 3.75 ± 0.15 (beta = −0.147) Habit to use the standing desk: IG mid > post mid vs. post: 3.59 ± 0.21 vs. 3.03 ± 0.21 (beta = −0.224) Subjective norm to use the standing: desk IG mid < post mid vs. post:4.11 ± 0.11 vs. 4.32 ± 0.11 (beta = 0.242) Relation with classmate: IG pre > post pre vs. post: 4.4 ± 0.2 vs. 4.2 ± 9.2 (beta = −0.04) Secondary schools Frequency of using the desks: IG mid < post mid vs. post: 1.30 ± 0.66 times/week vs. 1.42 ± 0.66 times/week (beta = 0.195) Pupils’ attitude towards the desks: IG mid < post mid vs. post: 3.71 ± 0.13 vs. 3.89 ± 0.13 (beta = 0.057)
Wick et al. (2018) [57]	Eriksen Flanker Task: Reaction time (congruous): IG pre > post pre vs. post: pre: 476 ± 99 ms vs. post: 451 ± 119 ms (*p* = 0.04) Accuracy: IG pre < post pre vs. post: pre: 0.87 ± 0.17% vs. post: 0.92 ± 0.16% (*p* = 0.01) Digit span task: Working memory: no significant Number of correct trials: no significant Cognitive function x group: no significant results	N/A	N/A
Cycling desk			
Fedewa et al. (2017) [30]	N/A	N/A	Mean value for all items: 3.87 ± 0.23/5 Preference to site on the bike compared to sitting in a chair: 4.63 ± 1.59/5 Sitting on the bike was fun: 4.13 ± 1.64/5
Ruiter et al. (2019) [58]	Digit Span Task: Accuracy and response times: no differences Visual Pattern Task: Accuracy and response times: no differences Eriksen Flanker Task: Responses time during incongruent: trials IG < CG (*p* = 0.01) Accuracy and response times: no differences Congruent and neutral stimuli: no differences Subjective task experience: Fatigue Motivation, difficulty, mental effort: no significant	N/A	N/A
Torbeyns et al. (2017) [34]	Rey auditory verbal learning test: no significant results Stroop test: Accuracy: on the word incongruent stimuli: IG post > pre pre vs. post: pre: 86.3 ± 10.9 ; post: 90.1 ± 7.1% (*p* = 0.030) Reaction time: IG and CG: pre > post pre vs. post: pre: 728.1 ± 105.6 ms vs. post: 694.2 ± 98.4 ms (*p* < 0.001 = Immediately repeated words: no significant Rosvold continuous performance test: Amplitude: post < pre post vs. pre: post: 3.2 ± 1.4 µV vs. pre: 3.9 ± 1.4 µV (*p* = 0.012) reaction time: no significant Attention during class: no significant Academic performance: no significant Mathematics test IG and CG post < pre post vs. pre: post: 0.39 ± 0.52 vs. pre: 0.66 ± 0.52 (*p* = 0.004)	N/A	N/A
Stability ball			
Erwin et al. (2016) [33]	On-task behaviors: no significant	N/A	N/A
Fedewa et al. (2015) [59]	Effect on-task behavior: On task: CG (87%) > IG (77%) Time working with peers: CG (15%) > IG (13%) Effect between Time and Group: (*p* < 0.01) pre < post (*p* < 0.01) Time doing independent work: CG (39%) > IG (29%) Effect time-group: (*p* = 0.02) pre < post (*p* < 0.01) Interaction time with the teacher: CG (33%) < IG (35%) Effect time-group: (*p* < 0.01) pre < post (*p* < 0.01) Effect on academic performance: Literacy: (IG + CG): post > pre (*p* < 0.01) Mathematics: (IG + CG): post > pre (*p* < 0.01)	N/A	N/A

N/A: Not applicable; NS: Not Significant; NR: Not Reported; IG: Interventional Group; CG: Control Group.

### 2.4. Data Collection

**Figure 1:**
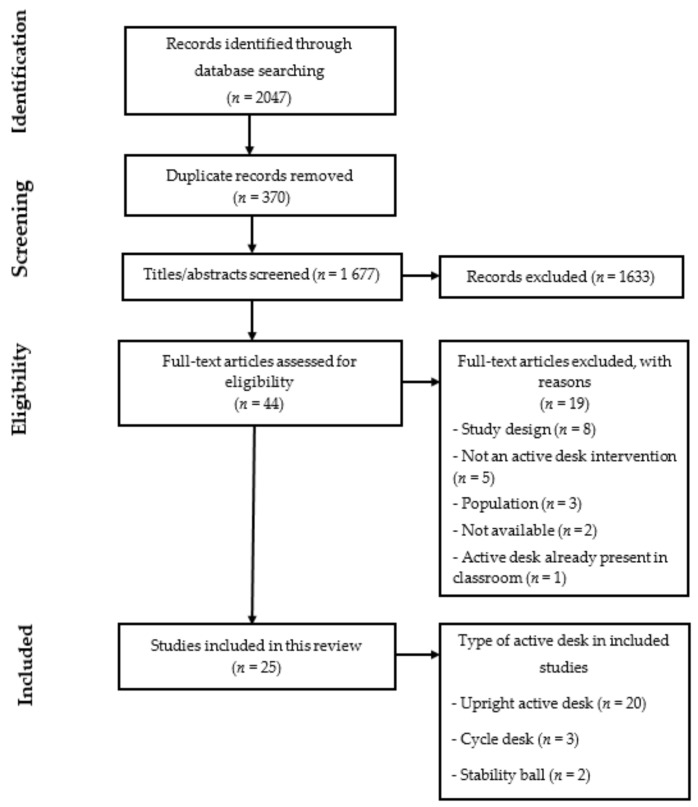
PRISMA flow chart.

Full texts from the articles were imported from a reference manager software (Zotero software; 5.0.21, CHNM, GMU, USA). After removal of duplicates, a screening was conducted by two independent authors on titles and abstracts to assess study eligibility (CC, TG). Identical procedure was used by the same authors on full text articles (CC, TG). Any disagreement regarding eligibility for inclusion was discussed until consensus emerged as made among the research team members. Each author completed data extraction files for every paper included. The process for trial inclusion is shown in the PRISMA flow chart (Figure 1).

### 2.5. Risk of Bias, Study Quality Assessment and Result Consideration

Risk of bias was independently examined by two authors (CC and TG) using the Cochrane risk of bias tool [60] (Table 5). Selection bias, performance bias, detection bias, attrition bias and reporting bias were assessed. The quality of evidence for each outcome by type of study design was determined using the Grading of Recommendations Assessment, Development and Evaluation (GRADE) framework [61] (Table 6). Any divergences were reported to the research team (MD, DT, LM). We did not exclude studies on the basis of risk of bias or low quality evidence. Importantly, the results of all the included studies and their directions, have been reported whether or not a statistical analysis was performed and if yes, précising whether the results reached or not the level of significance.

**Table 5:** Study risks of bias.

Studies	Random Sequence Generation	Allocation Concealment	Performance Bias	Detection Bias	Attrition Bias	Reporting Bias	Other Bias
Upright active desk							
Benden et al. (2011) [40]	Low risk	Unclear	High risk	High risk	Low risk	Low risk	Unclear
Benden et al. (2012) [41]	High risk	High risk	High risk	High risk	High risk	Low risk	Unclear
Benden et al. (2014) [42]	Low risk	Unclear	High risk	High risk	Low risk	Low risk	Low risk
Blake et al. (2012) [43]	High risk	High risk	High risk	High risk	High risk	High risk	Unclear
Clemes et al. (2016) (Bradford, England) [31]	High risk	Unclear	High risk	High risk	Low risk	Low risk	Unclear
Clemes et al. (2016) (Melbourne, Australia) [31]	Unclear	Unclear	High risk	High risk	Low risk	Low risk	Unclear
Clemes et al. (2020) [44]	Low risk	Low risk	High risk	High risk	Low risk	Low risk	Low risk
Dornhecker et al. (2015) [45]	Low risk	Unclear	High risk	High risk	Low risk	Low risk	Unclear
Ee et al. (2018) [32]	N/A	N/A	High risk	High risk	Low risk	Low risk	Low risk
Kidokoro et al. (2019) [46]	High risk	High risk	High risk	High risk	Low risk	Low risk	Low risk
Koepp et al. (2012) [47]	High risk	High risk	High risk	High risk	Low risk	Low risk	Unclear
Mehta et al. (2015) [48]	High risk	High risk	High risk	High risk	Low risk	Low risk	Low risk
Parry et al. (2019) [49]	N/A	N/A	High risk	High risk	Low risk	Low risk	Unclear
Pickens et al. (2016) [50]	High risk	Unclear	High risk	High risk	Low risk	Low risk	Unclear
Sherry et al. (2020) [51]	High risk	High risk	High risk	High risk	Low risk	Low risk	Low risk
Sprengeler et al. (2020) [52]	N/A	N/A	High risk	High risk	Low risk	Low risk	Low risk
Sudholz et al. (2016) [53]	High risk	High risk	High risk	High risk	Low risk	Low risk	Low risk
Swartz et al. (2019) [54]	N/A	N/A	High risk	High risk	Low risk	Low risk	Low risk
Verloigne et al. (2018) [55]	Low risk	Unclear	High risk	High risk	High risk	Low risk	Low risk
Wendel et al. (2016) [56]	Low risk	Low risk	High risk	High risk	Low risk	Low risk	Low risk
Wick et al. (2018) [57]	High risk	Unclear	High risk	High risk	Low risk	Low risk	Low risk
Cycling desk							
Fedewa et al. (2017) [30]	Low risk	Unclear	High risk	High risk	Low risk	Low risk	Unclear
Ruiter et al. (2019) [58]	Low risk	Low risk	High risk	High risk	Low risk	Low risk	Unclear
Torbeyns et al. (2017) [34]	Low risk	Unclear	High risk	High risk	Low risk	Low risk	Low risk
Stability ball							
Erwin et al. (2016) [33]	Low risk	Unclear	High risk	High risk	Low risk	Low risk	Unclear
Fedewa et al. (2015) [59]	Low risk	High risk	High risk	High risk	Low risk	Low risk	Unclear

N/A: not applicable; Other bias included any potential conflict of interest in studies.

**Table 6:** Association between the use of active desks and outcomes of included studies for the systematic review.

Outcome Assessment	No of Studies	Design	Quality Assessment					Quality
			Risk of Bias	Inconsistency	Indirectness	Imprecision	Other	
Body composition	School grade ranged between 1 and 10. Body composition was assessed objectively as body mass index, body mass, body fat percentage (bio-impedance), waist circumference.							
	5	RCT ^a^	No serious risk of bias	No serious inconsistency	No serious indirectness	Serious imprecision ^b^	None	MODERATE
	2	NRT ^c^	Serious risk of bias ^d^	Serious inconsistency ^e^	No serious indirectness	No serious imprecision	None	VERY LOW
Physical activity	School grade ranged between 1 and 12. Physical activity was assessed as light physical activity, MVPA, step counts, standing, stepping and walking time: by devices (accelerometers, inclinometers), self-reported questionnaires and/or external observations.							
	7	RCT ^f^	No serious risk of bias	Serious inconsistency ^g^	No serious indirectness	Serious imprecision ^h^	None	LOW
	6	NRCT ^i^	Serious risk of bias ^j^	No serious inconsistency	No serious indirectness	Serious imprecision ^k^	None	VERY LOW
	2	NRT ^l^	Serious risk of bias ^m^	No serious inconsistency	No serious indirectness	Serious imprecision ^n^	None	VERY LOW
	1	RT ^o^	No serious risk of bias	No serious inconsistency	No serious indirectness	No serious imprecision	None	LOW
	4	Cross-over ^p^	No serious risk of bias	No serious inconsistency	No serious indirectness	No serious imprecision	None	LOW
Sedentary behaviors	School grade ranged between 1 and 10. Sedentary behaviors were assessed by observations and/or self reporting questionnaires.							
	3	RCT ^q^	No serious risk of bias	No serious inconsistency	No serious indirectness	Serious risk of imprecision ^r^	None	MODERATE
	6	NRCT ^s^	Serious risk of bias ^t^	No serious inconsistency	No serious indirectness	Serious risk of imprecision ^u^	None	VERY LOW
	1	RT ^v^	No serious risk of bias	No serious inconsistency	No serious indirectness	No serious imprecision	None	LOW
	4	Cross-over ^w^	No serious risk of bias	No serious inconsistency	No serious indirectness	No serious imprecision	None	LOW
Energy expenditure	School grade ranged between 1 and 10. Energy expenditure was assessed by a portable device (armband) during school days and/or entire days.							
	4	RCT ^x^	No serious risk of bias	No serious inconsistency	No serious indirectness	Serious risk of imprecision ^y^	None	MODERATE
	1	NRCT ^z^	Serious risk of bias ^aa^	No serious inconsistency	No serious indirectness	Serious risk of imprecision ^ab^	None	VERY LOW
	1	NRT ^ac^	Serious risk of bias ^ad^	No serious inconsistency	No serious indirectness	Serious risk of imprecision ^ae^	None	VERY LOW
Cognitive and academic performance	School grade ranged between 2 and 10. Cognitive performance was assessed by cognitive functions test (working memory, inhibitory control, flexibility, attention) and on-task behaviors (observations, interviews)							
	4	RCT ^af^	Serious risk of bias ^ag^	No serious inconsistency	No serious indirectness	No serious imprecision	None	MODERATE
	4	NRCT ^ah^	Serious risk of bias ^ai^	No serious inconsistency	No serious indirectness	No serious imprecision	None	VERY LOW
	3	NRT ^aj^	Serious risk of bias ^ak^	No serious inconsistency	No serious indirectness	No serious imprecision	None	VERY LOW
Fatigue and musculoskeletal symptoms	School grade ranged between 4 and 10. Fatigue and musculoskeletal symptoms were assessed by questionnaires (Nordic Musculoskeletal Questionnaire, paper questionnaires), teacher observations, focus groups and/or interviews.							
	1	RCT ^al^	No serious risk of bias	No serious inconsistency	No serious indirectness	Serious imprecision ^am^	None	MODERATE
	2	NRCT ^an^	Serious risk of bias ^ao^	No serious inconsistency	No serious indirectness	Serious imprecision ^ap^	None	VERY LOW
	1	NRT ^aq^	Serious risk of bias ^ar^	No serious inconsistency	No serious indirectness	No serious imprecision	None	VERY LOW
	2	Cross-over ^as^	No serious risk of bias	No serious inconsistency	No serious indirectness	No serious imprecision	None	LOW
Process evaluation	Mean age ranged between 6 and 17 year. Perceptions and experiences of participants was assessed by questionnaires, self-reporting answers and/or interviews.							
	3	RCT ^at^	Serious risk of bias ^au^	Serious inconsistency ^av^	No serious indirectness	No serious imprecision	None	LOW
	4	NRCT ^aw^	Serious risk of bias ^ax^	No serious inconsistency	No serious indirectness	No serious imprecision	None	VERY LOW
Physical capacities and cardiometabolic health	School grade ranged between 4 and 10. Physical capacities was assessed by the 20 m shuttle run test. Cardiometabolic health was assessed by blood pressure.							
	2	RCT ^ay^	No serious risk of bias	No serious inconsistency	No serious indirectness	Serious imprecision ^az^	None	MODERATE

RCT: Randomized Controlled Trial; RT: Randomized Trial; NRCT: Non-Randomized Controlled Trial; NRT: Non-Randomized Trial. ^a^ Includes five randomized controlled studies [34,40,42,44,56]. ^b^ One study did not detail the age and sex of participants. Additionally, the results of body composition assessment were incomplete (the quality of evidence was downgraded from “high” to “moderate”). ^c^ Includes two non-randomized studies [41,47]. ^d^ Studies reported mixed findings (the quality of evidence was downgraded from “low” to “very low”). ^e^ Inconsistencies have been reported in the unit used in the results (the quality of evidence was downgraded from “low” to “very low”). ^f^ Includes seven randomized controlled studies [30,31,33,34,42,44,55]. ^g^ Inconsistencies have been reported in the number of participants (the quality of evidence was downgraded from “high to “moderate”). ^h^ Several studies did not achieve statistical analyses (the quality of evidence was downgraded from “moderate” to “low”). ^i^ Includes six non-randomized controlled studies [31,43,46,51,53,57]. ^j^ Studies reported mixed findings (the quality of evidence was downgraded from “low” to “very low”). ^k^ One study did not achieve statistical analyses (the quality of evidence was already at “very low”). ^l^ Includes two non-randomized studies [41,47]. ^m^ Studies reported mixed findings (the quality of evidence was downgraded from “low” to “very low”). ^n^ One study did not achieve statistical analyses (the quality of evidence was already at “very low”). ^o^ Includes one randomized study [50]. ^p^ Includes four cross-over studies [32,49,52,54]. ^q^ Includes three randomized controlled studies [31,44,55]. ^r^ One study did not achieve statistical analyses (the quality of evidence was downgraded from “high” to “moderate”). ^s^ Includes six non-randomized controlled studies [31,43,46,51,53,57]. ^t^ Studies reported mixed findings (the quality of evidence was downgraded from “low” to “very low”). ^u^ One study did not achieve statistical analyses (the quality of evidence was already at “very low”). ^v^ Includes one randomized study [50]. ^w^ Includes four cross-over studies [32,49,52,54]. ^x^ Includes four randomized controlled studies [30,34,40,42]. ^y^ One study did not achieve statistical analyses (the quality of evidence was downgraded from “high” to “moderate”). ^z^ Includes one non-randomized non-controlled study [43]. ^aa^ Studies reported mixed findings (the quality of evidence was downgraded from “low” to “very low”). ^ab^ One study did not achieve statistical analyses (the quality of evidence was already at “very low”). ^ac^ Includes one non-randomized study [41]. ^ad^ Studies reported mixed findings (the quality of evidence was downgraded from “low” to “very low”). ^ae^ One study did not achieve statistical analyses (the quality of evidence was already at “very low”). ^af^ Includes four randomized controlled studies [33,34,44,59]. ^ag^ One study did not achieve statistical analyses (the quality of evidence was downgraded from “high” to “moderate”). ^ah^ Includes four non-randomized controlled studies [43,45,51,57]. ^ai^ Studies reported mixed findings (the quality of evidence was downgraded from “low” to “very low”). ^aj^ Includes 3 non-randomized studies [47,48,58]. ^ak^ Studies reported mixed findings (the quality of evidence was downgraded from “low” to “very low”). ^al^ Includes one randomized controlled study [44]. ^am^ One study did not detail the number of participants for this outcome and did not achieve statistical analyses (the quality of evidence was downgraded from “high” to “moderate”). ^an^ Includes two non-randomized non-controlled study [51,53]. ^ao^ Studies reported mixed findings (the quality of evidence was downgraded from “low” to “very low”). ^ap^ One study did not achieve statistical analyses (the quality of evidence was already at “very low”). ^aq^ Includes one non-randomized study [47]. ^ar^ Studies reported mixed findings (the quality of evidence was downgraded from “low” to “very low”). ^as^ Includes two cross-over studies [32,49]. ^at^ Includes three randomized controlled studies [30,44,55]. ^au^ Studies reported mixed findings (the quality of evidence was downgraded from “high” to “moderate”). ^av^ Inconsistencies have been reported in the unit used in the results (the quality of evidence was downgraded from “moderate” to “low”). ^aw^ Includes four non-randomized controlled studies [43,46,51,53]. ^ax^ Studies reported mixed findings (the quality of evidence was downgraded from “low” to “very low”). ^ay^ Includes two randomized controlled studies [34,44]. ^az^ One study did not achieve statistical analyses (the quality of evidence was downgraded from “high” to “moderate”).

## 3. Results

### 3.1. Description of Studies

The PRISMA flow diagram presented in Figure 1 summarizes the study selection process. The search strategy initially found a total of 1677 references after removing duplicates. A total of 1635 articles were excluded after screening of titles and abstracts. Full text copies were obtained for 44 articles; of which 25 articles matched the inclusion criteria and were thus included in this systematic review. The main reasons for studies exclusion among the remaining were: (1) study design did not meet inclusion criteria (*n* = 8); (2) intervention did not use an active desk (*n* = 5); (3) population was not children without health issues (*n* = 3); (4) full texts were not available (*n* = 2); and (5) active desks were already integrated in classroom (*n* = 1). One article included two different study designs [31].

Ten studies were randomized controlled trials (RCT) [30,31,33,34,40,42,44,55,56,59] with four pilot studies [30,31,42,44]; seven were non-randomized controlled trials [31,43,45,46,51,53,57] with four pilot studies [31,51,53,57]; four were non-randomized trials [41,47,48,58] with two pilot studies [47,48]; one was a randomized trial [50] and four were crossover studies [32,49,52,54].

Among the included studies, 19 were conducted among primary school children aged 6–12 years [31,32,33,40,41,42,43,44,45,46,47,49,51,52,54,56,57,58,59], five took place among secondary level adolescents aged 12–17 years [30,34,52,56,58] and Verloigne et al. [55] enrolled children in both levels aged 10–16 years.

Two studies included boys only [32,49], three did not specify the gender [30,42,48] and the rest of the studies included both boys and girls [31,33,34,41,42,44,45,46,47,48,50,51,52,53,54,55,56,57,58,59].

Twenty studies assessed upright active desks (i.e., standing desk, sit-to-stand desk and stand-biased desk) [31,32,40,41,42,43,44,45,46,47,48,49,50,51,52,53,54,55,56,57]; three cycle desks [30,34,57] and two used stability balls [33,59]. Active desks have been described in Table 1.

In twenty-one studies, active desks were allocated to every individual [31,32,33,34,40,41,42,43,46,47,48,49,50,51,52,53,54,56,57,58,59]. Verloigne et al. [55] implemented three standing desks per classroom, Clemes et al. [44] provided six active desks in each class and Fedewa et al. [30] provided four active desks in interventional group. One study did not specify the number of implemented active desks [45].

All studies had an intervention duration from two weeks to two years. Verloigne et al. [55] suggested a rotation every half class while Clemes et al. [31] recommended to use active desks at least 30 min per day (Australian study) and one hour per day (English study). Some studies suggested also to practice active desks at least one hour per day [44,57] or for four class hours of 50 min per week [34]. Several studies did not indicate the active desks time and frequency use [47,49,51,58,61]. Some interventions enabled active desks to be free to use [30,40,41,42,43,46,47,48,52,53] or to use it for the entire school day [32,33,45,59]. In one study, active desks were only used for the evaluations [58].

### 3.2. Data Synthesis by Outcome

#### 3.2.1. Body Composition

Six studies assessed body composition when using upright active desks [40,41,42,44,47,56] and one with cycling desks [34]. However, one study did not detail their results on this outcome [40] (Table 6). Wendel et al. [56] found a significant difference in BMI for interventional group compared to the control group after two years of intervention (−5.24 for BMI percentile) (Table 3). Other studies did not report any change in BMI with the use of an upright active desk.

Torbeyns et al. [34] observed a significant effect of time for height, body weight, fat mass percentage and waist circumference without condition effect. However, traditional desks group reported a significantly higher BMI while cycling desks group did not find any difference.

#### 3.2.2. Sedentary Behaviors

Thirteen articles using upright active desks assessed sedentary behaviors [31,32,43,44,46,49,50,51,52,53,54,55,57], while only one used cycling desks [30]. As presented in Table 3, two studies observed that children, when using upright active desks, spent significantly less time sedentary than the control group, using objective measurements [46,54]. Other studies did not find any difference for the interventional group [49,53]. Moreover, Ee et al. [32] observed no significant difference for whole day sedentary time but reported a significant reduction in sitting time during school hours for the intervention group compared to the control group. Similar results have been reported in four other articles [31,44,52,53]. Additionally, four studies reported a reduction of sitting time between T0 and T1 for the intervention group [44,46,51,58]. Similar results have been found in another study but were not statistically significant [49,52].

Fedewa et al. [30] reported a decreased of 9.5% in sedentary time for the intervention group compared to the control group.

#### 3.2.3. Physical Activity

Sixteen articles assessed physical activity using upright active desks [31,32,41,42,43,44,46,47,49,50,51,52,53,54,55,57], two with cycling desks [30,34] and one with stability balls [33]. Studies assessing the upright active desk effects on physical activity reported several different outcomes such as light physical activity, moderate-to-vigorous physical activity (MVPA), step counts, stepping, standing and walking time (Table 3).

For light physical activity, four studies reported no significant changes for interventional group compared to the control group [32,50,52,59,61]. For MVPA, studies found contradictory results while two studies did not find any change [32,49]. Kidokoro et al. [46] observed a significant increase in MVPA for the intervention group between pre- and post-intervention. Another study [54] found that MVPA decreased for the intervention group during school years but less than the control group. Additionally, they reported that the benefit of upright active desk was greater among students initially determined as less active.

Statistically significant increases were reported for the intervention group standing time in height studies [31,32,43,44,50,51,53,55,57]. Similar results have been reported but without reaching significance [49,52].

Regarding step counts, Benden et al. [41] reported an increase of this outcome without statistical analyses (Table 3 and Table 6). In another study, they reported similar results at mid intervention but not at the end [42]. No significant effects were observed in two other studies [47,50]. In the article of Clemes et al. [31], the study in Australian school reported no significant effect while the British ones showed an increase for the intervention group in post intervention.

For stepping time a significant decrease was reported for the intervention group [55] or no effect [31,57]. One study [51] observed a significant increase while Clemes et al. [44] found similar results but no statistical analyses have been reported.

Torbeyns et al. [34] assessed the effect of cycling desks on physical activity with a questionnaire. Interventional group and control group decreased their physical activity time between pre- and post-intervention but no condition effect was observed. Despite the lack of statistical analyses (Table 2 and Table 4), one study reported, with an objective measurement, an increase of light physical activity and MVPA for the intervention group compared to the control group [30].

One study using stability balls assessed physical activity and missed to observe any difference between the interventional group and the control group after the intervention [33]. Additionally, all groups decreased their physical activity level and their step count between pre- and post-intervention.

#### 3.2.4. Energy Expenditure

Four studies assessed energy expenditure with the use of upright active desks [42,43,48,54] and two with cycling desks [30,34]. All upright active desks studies observed an increase between 15% and 25.7% in energy expenditure for interventional groups compared to control groups [42,43,48,54] (Table 2).

Cycling desk studies reported also an increase of energy expenditure. Torbeyns et al. [34] showed a significant increase in energy expenditure (36%) using cycling desks compared to traditional desks. Fedewa et al. [30] reported similar results without any statistical analyses (Table 3 and Table 6).

#### 3.2.5. Physical Capacities and Cardiometabolic Health

Physical capacities were only evaluated in one study that used cycling desks [34]. The authors reported an increase in the performance during the 20 m shuttle run test in their interventional group compared to the control group (+0.6 interval) (Table 3). Moreover, there was a significantly lower rate of perceived exertion (RPE) in the interventional group compared to the control group after 22 weeks. For cardiometabolic health, only Clemes et al. [44] assessed blood pressure with the use of an upright active desks. They reported an increase in systolic blood pressure in the interventional group but the authors did not perform statistical analyses (Table 2 and Table 4).

#### 3.2.6. Cognitive and Academic Performance

Seven studies assessed cognitive and academic performance when using upright active desks [44,48,49,51,53,55,56], two studies with cycling desks [34,58] and two with stability balls [33,59]. Concerning executive functions (working memory, inhibitor control, cognitive flexibility), visual working memory was assessed in two studies using upright active desk and two studies using cycling desks and no change was reported [34,53,56,57]. As detailed in Table 4, inhibitory control has been assessed in three studies, and the use of cycling desks shown to significantly increase the inhibitor control in the intervention group compared to the control group with an higher increase of accuracy for the intervention group (4.21%) [58]. One of the studies that used upright active desk reported an improvement in both reaction time and accuracy [57] while the other reported no significant change [48]. The reaction time for cognitive flexibility decreased after intervention in the study that used upright active desks [57]

Regarding to academic engagement and attention, two studies using upright active desks reported an increase in the intervention group compared to the control group [43,45] without any change in concentration and classroom management [33,44,55]. A study using stability balls reported more interaction time with teachers but the time working with other students or independently were reduced compared to the control group after intervention. Both groups observed improvement in mathematics and literacy but they were not related specifically to the intervention [59]. Mehta et al. [48] assessed several outcomes where they primarily observed a significant increase in cognitive performance with the use of upright active desks compared to traditional ones.

#### 3.2.7. Fatigue and Musculoskeletal Pain Symptoms

Six studies, all with upright active desks, assessed fatigue and musculoskeletal pain symptoms [32,44,51,52,55,59]. Three studies reported no difference on those outcomes between upright active desks and traditional desks [44,51,55]. Significant changes have been reported in two studies [32,49] with a decrease of pain symptoms in the neck and shoulder area. Nonetheless, a study observed that 51% of children have experienced pain in legs and back area with the use of upright active desks [53] (Table 4).

#### 3.2.8. Process Evaluation

Acceptability and feasibility have been assessed in several studies [30,43,44,46,51,53,55]; one was cycling desks [30] and others were upright active desks. One study reported retention rates of 100% for schools and 97% for children with an overall recruitment rate at 33% [44] (Table 4). Studies have shown a good acceptability of upright active desks in children [48,50,51], with a willingness to use it in the future and a reduction of sleepiness [46]. From teachers’ perspective, they have declared a positive influence of upright active desks to complete tasks and are willing to continue teaching with upright active desks [53]. One study reported that parents have felt a positive impact on their children’s behavior at school [43]. However, one study [55] reported some negative effects with the use of upright active desks such as a slight deterioration of the relation with classmates. Authors also reported, a decrease of the mean duration and habit to use upright active desks over time. Most of those observations were reported in primary schools; secondary schools observed an improvement of the attitude towards the desk [55].

For cycling desks, authors [30] observed no change in attention and task completion compared to traditional desks. Students also experimented a reduction of fidgeting. Their preference to sit on cycling desks compared to traditional desks was higher despite the lack of a comfortable seat. Overall, cycling desks have been perceived by teachers and students as a positive tool to improve the environment of school class.

It was determined by the review team that a meta-analysis was not possible due to high levels of heterogeneity across studies; narrative syntheses were employed instead. The overall quality of the included studies was low due to methodological inconsistencies, in addition of the heterogeneity in terms of statistical and clinical characteristics (Table 5 and Table 6).

## 4. Discussion

We are currently at a time where sedentary behaviors are a worldwide concern and classroom active desks have been proposed as a potential solution to counterbalance their adverse effects on health-related outcomes. Several reviews evaluated the effect of some specific types of active desks [35,36] on some specific outcomes such as academic achievement and cognitive outcomes [37]. The present work is the first systematic analysis of the existing literature on active desk implementation in the school environment and their effects on physical activity, sedentary behavior, academic achievements and overall health. According to our results, (i) cycling desk may be a promising active desk to increase physical activity while reducing sedentary behaviors; also, cycling desk is associated with positive cognitive performance and is well-received in the school environment; (ii) studies need to better identify and detail their active desks use; (iii) further studies have to use stronger methodologies to enable comparisons and conclusions regarding the real effects of each active desks.

Among all the included studies that assessed body composition, little or none effect was observed from the use of upright active desks or cycling desks. The only study that found positive changes in body composition was the study that lasted 2 years with upright active desks [56]. This suggests that the time of exposure to active desks can be an important parameter to consider. Additionally, the lack of observed effect on body composition in the reviewed studies can be potentially explained by the low level of energy expenditure generated by active desks. While active desks substantially increase students’ energy expenditure compared to traditional desks [30,34,42,43,48,54], the magnitude of responses may not be sufficiently important to induce significant changes in body composition. However, it is important to notice that the range of increase in energy expenditure is not the same across active desks, with cycling desks generating a higher energy expenditure compared with upright active desks. According to our analysis, active desks also seem to positively influence sedentary behaviors. Indeed, by using upright active desks, students spend more time in a standing position and less time seated. Even though “standing” is not included in the definition of sedentary behavior [4], the energetic cost of this passive posture can be under 1.5 METs [62] and this long-term position can be a potential source of musculoskeletal pain [63]. From that perspective, replacing traditional desks by active desks (maybe not only standing), which increase energy expenditure, may be promising due to the replacement of a sitting time to an active behavior. Concerning cognitive and academic performance, all studies reported either no change or an improvement in students, leading to consider the non-deleterious impact of active desks on cognition. This finding is particularly relevant, as the implementation of active desks is clearly dependent on the willingness of the academic actors and parents. Beyond the cognitive aspect, active desks were well received by students and teachers in most studies, suggesting the possibility that active desks can be easily implemented in the school setting.

### Methodological Concerns

The tremendous amount of sitting time spent in classrooms led scientists to examine how active desks for children and adolescents can be used to reduce sedentary behaviors. There has been a constant increase of studies focusing on this target in recent years. However, by systematically reviewing the current literature on the topic, we observed several methodological issues. The lack of strong and reliable results did not allow us to perform any meta-analysis to avoid misleading errors [64]. Not only the lack of methodological consistency between studies is concerning but also the relatively low quality of the included works (Table 5 and Table 6) are certainly the main conclusions emerging from the present systematic review.

Indeed, although there is an increasing number of RCT on the topic in the scientific literature, only 10 of the 23 included studies were RCT in the present analysis. The heterogeneity of designs makes any comparison difficult regarding the potential benefits of active desks on health. Similarly, the variety of methods used to evaluate similar parameters (e.g., evaluation of physical activity and/or sedentary behaviors using accelerometers or inclinometers or questionnaires and expressed as counts, vector of magnitudes, activity or standing/sitting time for instance) prevent any strong collective evidence.

As previously suggested in the literature [65], studies should use parameters indicated in the last available recommendation to evaluate sedentary behaviors, sedentary time and physical activity. Moreover, studies assessing sedentary behaviors and physical activity should also consider the recording time for valid and reliable data. In other words, to observe and understand behavioral changes and compensatory effects, studies should not only record data during class time but rather on overall days, school and non-school day.

Importantly, while the recent years have seen a growing number of studies implementing active desks at school at a time where it was necessary to adopt new solutions and strategies to counteract the adverse effects of sitting time, these studies missed to clearly detail the exact conditions of use of their active desks. Indeed, as underlined in our systematic analysis, most of the included studies do not provide details regarding the time of use of their active desks, the instructions given to the teachers and pupils, which once more, make any practical recommendation hazardous. Characterization of the workload and details regarding the practical instructions should be a priority for investigators to understand at which frequency, intensity and duration active desks are driving benefits or adverse effects. In addition, it appears even difficult to clearly understand which kind of active desk has been used when reading some studies. Indeed, while some studies claim to use standing desks and formulate recommendations and conclusions regarding the use of the standing position at school, it appears that some stools are provided with each desk and that the exact time spent standing is not evaluated or even presented. Literally, it may be possible that users can sit or recline on the stool most of their class time. Considering this information, there is a clear risk of misunderstanding by using the term standing desk when it refers to a stand-biased desk. To avoid any misconception, the following definitions for those three active desks are proposed: (i) standing desk: desk which enables users to be in a standing position, without allowing any support to sit or recline; (ii) sit-to-stand desk: desk enabling users to switch from a sitting to a standing position at their discretion by adjusting the desk height; (iii) stand-biased desk: desk which enables users to be in a standing position while having a support such as a stool to sit or recline at their discretion.

Upright active desks represent a majority of the included study, while cycling desks and stability balls represent only five studies. Regarding results obtained in adults, cycling desk is suggested as the best compromise between all active desks [66] but not enough studies have assessed its effects in children and adolescents. While further studies are needed in the pediatric population, we also encourage future investigations to consider the effects of such active desks on physical discomfort, cognitive performance, physical capacities or physiological components that remain underexplored. Similarly, some ergonomic and process evaluations should be considered, which would benefit for a better use and implementation of these desks. Effectively, whether active desks are showing positive effects on several outcomes, one priority remains to understand if they are well accepted in school class by children, teachers and parents.

Furthermore, studies are essentially focusing on primary schools (17 of the 23 studies included). As previously said, children at 15 years spend on average 75% of their waking day in a sitting position [13]. Then, there is obviously a lack of active desks implementation in secondary school. From this perspective, scientists should also consider secondary levels. Therefore, to better understand the effect of active desks, further investigations should focus on large sample RCT follow-up in primary and secondary level (long-term follow-up), assessing multicomponent outcomes with valid, reproducible and reliable methods, while quantifying the workload. There is a need for a better description of the active desks use and condition of its use to avoid any misconception and inaccuracies. Additionally, scientists must consider the feasibility and the implementation of active desks in the school environment.

## 5. Conclusions

Active desks appear as a promising tool to reduce sedentary behaviors in school environment. In the present state of knowledge, the effects of all active desks appear not equivalent, mainly due to the difference in body activation and energy expenditure. Regarding the relatively low number of available studies and the high degree of heterogeneity in terms of quality, design and methods, comparisons and conclusions remain difficult at the moment. The present systematic analysis calls for further well-designed studies to better understand the effects of the use of active desks among children and adolescents in order to inform policy and practice.

## Data Availability

Not applicable.
